# Zinc, Iron, Manganese and Copper Uptake Requirement in Response to Nitrogen Supply and the Increased Grain Yield of Summer Maize

**DOI:** 10.1371/journal.pone.0093895

**Published:** 2014-04-04

**Authors:** Yanfang Xue, Shanchao Yue, Wei Zhang, Dunyi Liu, Zhenling Cui, Xinping Chen, Youliang Ye, Chunqin Zou

**Affiliations:** 1 Center for Resources, Environment and Food Security, China Agricultural University, Beijing, China; 2 College of Resources and Environmental Sciences, Henan Agricultural University, Zhengzhou, China; Auburn University, United States of America

## Abstract

The relationships between grain yields and whole-plant accumulation of micronutrients such as zinc (Zn), iron (Fe), manganese (Mn) and copper (Cu) in maize (*Zea mays* L.) were investigated by studying their reciprocal internal efficiencies (RIEs, g of micronutrient requirement in plant dry matter per Mg of grain). Field experiments were conducted from 2008 to 2011 in North China to evaluate RIEs and shoot micronutrient accumulation dynamics during different growth stages under different yield and nitrogen (N) levels. Fe, Mn and Cu RIEs (average 64.4, 18.1and 5.3 g, respectively) were less affected by the yield and N levels. ZnRIE increased by 15% with an increased N supply but decreased from 36.3 to 18.0 g with increasing yield. The effect of cultivars on ZnRIE was similar to that of yield ranges. The substantial decrease in ZnRIE may be attributed to an increased Zn harvest index (from 41% to 60%) and decreased Zn concentrations in straw (a 56% decrease) and grain (decreased from 16.9 to 12.2 mg kg^−1^) rather than greater shoot Zn accumulation. Shoot Fe, Mn and Cu accumulation at maturity tended to increase but the proportions of pre-silking shoot Fe, Cu and Zn accumulation consistently decreased (from 95% to 59%, 90% to 71% and 91% to 66%, respectively). The decrease indicated the high reproductive-stage demands for Fe, Zn and Cu with the increasing yields. Optimized N supply achieved the highest yield and tended to increase grain concentrations of micronutrients compared to no or lower N supply. Excessive N supply did not result in any increases in yield or micronutrient nutrition for shoot or grain. These results indicate that optimized N management may be an economical method of improving micronutrient concentrations in maize grain with higher grain yield.

## Introduction

Maize (*Zea mays* L.), as one of the world's leading cereal grains along with rice and wheat, is very popular due to its diverse functionality as a food source for both humans and animals [1]. It is estimated that maize together with rice and wheat provide at least 30% of the food calories to more than 4.5 billion people in 94 developing countries [2]. Increased maize production is also required to meet the demands for animal feed and biofuel [3].

In China, maize accounts for more than one-third of Chinese cereal production and China is responsible for 20.9% of global maize output from 2009 to 2011 [4]. Pursuing high grain yields in China has been the top priority in policy. However, a 71% increase in total annual grain production from 1977 to 2005 was accompanied by a 271% increase in nitrogen (N) fertilizer input, which resulted in serious environmental problems such as eutrophication, greenhouse gas emissions and soil acidification in China [5]–[8]. Intensive cultivation of high-yielding cultivars with over-applications of N, phosphorous (P) and potassium (K) fertilizers also leads to micronutrient, especially zinc (Zn) and iron (Fe) deficiencies in many countries [9]. For example, in China, approximately 40% of the soils are Zn and Fe deficient, and about 30% are manganese (Mn) and copper (Cu) deficient [10]. Therefore, improving N management and developing related policies are major issues in crop production and environmental protection in China. Several studies have reported that increasing N fertilization has little or no effect on grain micronutrient concentrations, such as Zn, Fe and Cu in maize [11]–[13]. Therefore, it is necessary to investigate whether optimal N management by decreasing excessive N supply, would result in negative impacts on micronutrient nutrition of maize plants and their allocation into grains under field conditions.

Maize grain yield has steadily increased over the last century through both conventional breeding and agronomic practices [14]. Increased grain yield and biomass production may be associated with greater uptake of total plant nutrients, such as N, P and K [15], [16]. A double peak and a higher N and P accumulation rates in maize with higher yield have been found compared to lower yielding maize [15]. Little information is available about the accumulation dynamics of micronutrients such as Zn, Fe, Mn and Cu in response to an increased grain yield or increased biomass production. However, the patterns of micronutrient accumulation during different growth stages have been quantified [17], [18]. It has been suggested that further investigation is needed to understand the timing of micronutrient uptake in response to yields, which varies between crop species, environments and management practices [19]. Knowledge of the dynamics of micronutrient accumulation associated with yield–trait relationships in crops would provide an efficient tool to synchronize micronutrient demand and supply, thus improving nutrient management efficiencies (e.g. Zn fertilizer) and benefiting sustainable production without harming the environment.

The study of the nutrient reciprocal internal efficiencies (RIEs, g of nutrient requirement in plant dry matter per Mg grain yield) could be used to determine the relationships between grain yield and whole-plant nutrient accumulation [19]. Some studies have reported large variations in the relationship between maize grain yield and nutrient accumulation in response to nutrient supplies, grain yields, genotypes, and environments [20]–[23]. For example, N reciprocal internal efficiency (N requirement per Mg of grain yield) decreased from 19.8 to 16.9 kg with increasing grain yield while it was higher with N application than no N application [22]. These studies mainly focused on N, P and K. Little information is available on micronutrient accumulation (such as Zn, Fe, Mn and Cu) in response to different grain yields, N fertilization rates or different cultivars. Understanding the relationships between micronutrient accumulation, especially Zn, and grain yield with different N management practices should provide valuable information leading to improvements in micro-fertilizer management practices. It is reported that crop recovery efficiency of micronutrients ranged from only 5% to 10% [24].

Recent studies have shown that increased maize grain yield decreases grain N concentration [14], [22]. A negative correlation between grain yield and concentrations of Zn and Fe in maize grain has also been reported [25]. Therefore, it is important to investigate the concentrations of Zn and Fe, together with Mn and Cu, in maize grain in response to increased grain yield.

The objectives of this study were to (i) quantify RIEs (ZnRIE, FeRIE, MnRIE and CuRIE) in relation to increased grain yield, different cultivars and N management practices, (ii) estimate shoot Zn, Fe, Mn and Cu accumulation dynamics in response to increased grain yields and different N management practices, and (iii) investigate the effects of improved grain yields, different cultivars and N management practices on the concentrations of Zn, Fe, Mn and Cu in maize grain.

## Materials and Methods

### Experimental design

Field experiment I was conducted in four consecutive years (2008–2011) in Quzhou county (36°53′60″ N, 115°0′ E) in Hebei province, on a calcareous alluvial soil typical of the North Plain of China (NCP), pH 8.3 (1∶2.5 w/v in water) and 1.4% organic matter [26]. One cultivar (Zhengdan958) of maize was planted in a randomized complete block design with four replicates (300 m^2^ plot^−1^) in 2008 and 2009 while three maize cultivars (Zhengdan958, Xianyu335 and Yedan13, recorded as ZD, XY, YD, respectively) were planted in 2010 and 2011 in split-plots (N fertilizer as the main plots and cultivars as sub-plots) with three or four replicates. Nitrogen rates were applied as urea as follows: no N application (recorded as N-0), 40–70% of optimized N treatment (recorded as N-low), optimized N treatment (240, 150, 105 and 193 kg N ha^−1^ in four consecutive years, respectively, recorded as N-opt) based on the in-season root-zone N-management approach, as previously reported [3], [27], and 130 or 150% of optimized N treatment and farmer's nitrogen practice (250 kg N ha^−1^, recorded as N-over). Nitrogen fertilizer was applied in split doses as described in Table S1. Before sowing, 45 kg ha^−1^ of P_2_O_5_, 45 kg ha^−1^ of K_2_O, and 30 kg ha^−1^ of ZnSO_4_·7H_2_O (without application of ZnSO_4_·7H_2_O in 2011) were broadcast and incorporated into the upper 0–15 cm of the soil by rotary tillage. Another 45 kg ha^−1^ of K_2_O was applied by hand as a top dressing at the V10 (ten-leaf) growth stage. The climate conditions for the experiment have been recently reported by Zhang et al. [28].

Field experiment II was conducted in 2009 in Wenxian (34°52′42″ N, 112°57′29″ E), Henan province in the middle of the NCP. One maize cultivar (Fengyu 4, recorded as FY) was planted in a randomized complete block design with three replicates (20 m^2^ plot^−1^). Nitrogen rates were applied as urea as follows: no N application (recorded as N-0), 50% and 75% of optimized N application (recorded as N-low), optimized N application (240 kg N ha^−1^, recorded as N-opt) and 150% of optimized N application (recorded as N-over). Nitrogen fertilizer was applied in split doses as described in Table S1. In addition, 90 kg ha^−1^ of P_2_O_5_ and 90 kg ha^−1^ of K_2_O were applied by hand at the V5 (five-leaf) growth stage.

The two experiments were conducted in a winter wheat-summer maize rotation system. Nitrogen fertilizer, P_2_O_5_ and K_2_O were applied as urea, superphosphate and potassium chloride, respectively. Weeds were well controlled, and no obvious water or pest stress was observed during the maize growing season. No specific permissions were required for these locations. The field studies did not involve endangered or protected species.

### Sampling and nutrients analysis

6-plant (experiment I) and 2-plant (experiment II) samples were collected at V6 (six-leaf), V12 (twelve-leaf), R1 (silk emerging), R3 (milk stage) and R6 (physiological maturity, divided into straw and grain) growth stages. All plant samples were collected at V6, V12, R1, R3 and R6 stages in experiment I in 2008 and 2009. In 2010, plant samples were only collected at R1 and R3 stages with three N treatments (no N application, optimized N application and farmer's nitrogen practice) and at R6 with five N treatments. In 2011, plant samples were only collected at V6 with one cultivar (ZD) and N treatments excluding 130% of optimized N treatment, at R1 with three cultivars (ZD, XY and YD) and three N treatments (no N application, optimizer N application and farmer's nitrogen practice) and R6 stages with three cultivars (ZD, XY and YD) and five N treatments. All plant samples were collected at V6, V12, R1, R3 and R6 stages in experiment II. The shoots were rapidly washed with deionized water and then oven-dried at 70°C to determine dry weight. Plant samples were ground with a stainless steel grinder (RT-02B, Taiwan, China) and digested with HNO_3_-H_2_O_2_ in a microwave accelerated reaction system (CEM, Matthews, NC, USA). The concentrations of Zn, Fe, Mn and Cu in the digested solutions were determined by inductively coupled plasma atomic emission spectroscopy (ICP-AES, OPTIMA 3300 DV, Perkin-Elmer, USA). IPE556 grain and IPE883 straw (Wageningen University, The Netherlands) were used as reference materials.

To estimate grain yields, ears in the central part of 60 or 180 m^2^ (experiment I) and 10 m^2^ (experiment II) areas were harvested at maturity. At harvest, sub-samples of 6 plants in the two experiments were collected and divided into grain and straw parts to determine above-ground biomass and the harvest index (HI) after shoots were oven-dried at 70°C.

### Data analysis

According to grain yield, all data collected from 2008 to 2011 were divided into four yield ranges: <7.5, 7.5–9, 9–10.5 and >10.5 Mg ha^−1^. According to these yield ranges, maize biomass and shoot micronutrient (Zn, Fe, Mn and Cu) accumulation as well as micronutrient RIEs (ZnRIE, FeRIE, MnRIE and CuRIE) were analysed. For each of the four different cultivars (YD, XY, ZD and FY), RIEs were analysed. According to N management practices, all data were also divided into four groups: N-0 (no N application), N-low (30–75% of optimized N treatment), N-opt (optimized N treatment) and N-over (130% or 150% of optimized N treatment as well as farmer's nitrogen practice). The number of observations for shoot samples at different growth stages with different yield ranges and N levels was shown in Table S2. All the above data analysis referred to others [22], [29], [30]. The following parameters were calculated: Micronutrient harvest indices (ZnHI, FeHI, MnHI and CuHI)  =  grain micronutrient accumulation/shoot micronutrient accumulation (1); Grain micronutrient accumulation  =  grain micronutrient concentration x grain dry weight (2); Shoot micronutrient accumulation  =  shoot micronutrient concentration x biomass (3); Micronutrient RIE  =  shoot micronutrient accumulation/grain yield  =  grain micronutrient accumulation/(grain yield x micronutrient harvest index) [16], [31] (4).

The Pearson correlation procedure was used to evaluate the correlations among the measured parameters of all data using SAS software (SAS 8.0, USA). Means of different yield ranges and N levels were compared using one-way ANOVA at a 0.05 level of probability followed by Duncan test of SPSS 13.0 for Windows. All results were expressed on a dry weight basis with an exception of grain yields. Grain yields were reported at standard moisture of 15.5%.

## Results

### Grain yields, yield components, biomass production and micronutrient (Zn, Fe, Mn and Cu) accumulation in shoot and grain

Overall, maize grain yield (n = 149) averaged 8.0 Mg ha^−1^ with a range from 3.9 to 12.8 Mg ha^−1^ (Table 1). The total biomass production at physiological maturity averaged 13.2 Mg ha^−1^, with a harvest index (HI) of 51% (Table 2). The Zn, Fe, Mn and Cu RIEs averaged 31.0, 64.4, 18.1, and 5.3 g, respectively. The concentrations of grain Zn, Fe, Mn and Cu averaged 16.3, 15.8, 3.2 and 1.4 mg kg^−1^, respectively. The harvest indices of Zn, Fe, Mn and Cu averaged 48%, 23%, 16% and 24%, respectively (Table 2). Yield was significantly negatively associated with grain Zn concentration (r = −0.36***) but positively associated with grain Mn concentration (r = 0.22**). However, yield was not correlated with grain concentrations of Fe and Cu (Table 3).

**Table 1:** Descriptive statistics of yield for total samples, four yield ranges (15.5% moisture), four different cultivars and four nitrogen (N) levels.

	n^a^	Mean	SD^b^	Minimum	25% Q^c^	Median	75% Q	Maximum
		----------------------------------Mg ha^−1^------------------------------						
Total	149	8.0	2.1	3.9	6.4	8.1	9.5	12.8
Yield ranges (Mg ha^−1^)								
<7.5	58	5.9	1.0	3.9	5.2	5.9	6.7	7.4
7.5–9	45	8.2	0.4	7.5	7.8	8.3	8.5	9.0
9–10.5	26	9.7	0.4	9.0	9.2	9.6	10.0	10.4
>10.5	20	11.4	0.6	10.5	11.0	11.3	11.8	12.8
Cultivars								
YD	30	5.6	1.1	3.9	4.7	5.4	6.5	8.1
XY	30	8.0	1.5	3.9	7.3	8.4	9.1	10.8
ZD	75	8.5	1.9	5.0	7.2	8.3	9.8	12.8
FY	14	10.2	1.2	8.1	9.5	10.4	11.0	12.0
N levels								
N-0	30	6.1	1.5	3.9	5.1	5.8	6.7	9.5
N-low	32	8.3	2.1	4.3	7.3	8.2	9.8	11.5
N-opt	30	8.9	2.1	4.4	7.4	9.2	10.4	12.3
N-over	57	8.3	1.8	4.5	7.2	8.3	9.3	12.8

n^a^: number of observations.

SD^b^: standard deviation.

Q^c^: quartile.

**Table 2:** Biomass production, yield components (including ears number, grains per ear and thousand grain weight (TGW)), harvest index (HI), micronutrient reciprocal internal efficiencies (ZnRIE, FeRIE, MnRIE and CuRIE), grain micronutrient concentrations (GZnC, GFeC, GMnC and GCuC), straw micronutrient concentrations (SZnC, SFeC, SMnC and SCuC) and micronutrient harvest index (ZnHI, FeHI, MnHI and CuHI) of summer maize as affected by different yield ranges, cultivars and N levels.

Parameters	Total	Yield ranges (Mg ha^−1^)				Cultivars				N levels			
		<7.5	7.5–9	9–10.5	>10.5	YD	XY	ZD	FY	N-0	N-low	N-opt	N-over
	n^a^ 149	58	45	26	20	30	30	75	14	30	32	30	57
Biomass (Mg ha^−1^)	13.2	10.2d	14.0c	14.9b	17.5a	9.7c	14.2b	13.5b	16.4a	10.3b	13.9a	14.5a	13.6a
Ears number (10^4^ ha^−1^)	7.4	7.3a	7.5a	7.5a	7.5a	6.8b	7.8a	7.9a	5.4c	7.5a	7.3a	7.3a	7.6a
Grains per ear	425	379c	420b	477a	500a	429b	352c	416b	616a	352b	444a	450a	439a
TGW (g)	275	257b	288a	283a	292a	233c	314a	277b	277b	268a	276a	277a	278a
HI (%)	51	50c	50c	53b	55a	49b	48b	53a	53a	50a	51a	52a	51a
ZnRIE (g)	31.0	36.3a	32.4a	26.6b	18.0c	41.0a	36.1b	26.6c	22.0d	28.9a	28.4a	31.2a	33.4a
GZnC (mg kg^−1^)	16.3	16.7a	17.4a	16.6a	12.2b	17.6a	16.0a	16.4a	13.6b	15.2b	15.2b	16.6ab	17.3a
SZnC (mg kg^−1^)	20.0	24.4a	20.2b	17.1b	10.7c	29.0a	23.7b	16.1c	13.8c	18.2ab	17.6b	20.9ab	21.8a
ZnHI (%)	48	41d	48c	54b	60a	37b	40b	55a	53a	48a	49a	49a	48a
FeRIE (g)	64.4	68.6a	64.6a	59.7a	57.4a	66.8b	54.4c	61.2bc	97.4a	65.8a	69.0a	59.9a	63.4a
GFeC (mg kg^−1^)	15.8	15.9a	16.2a	16.6a	13.8b	17.6a	13.9b	15.9a	16.0a	13.4c	15.5b	16.0ab	17.2a
SFeC (mg kg^−1^)	62.6	63a	62a	61a	66a	58.6b	46.6c	61.9b	109.4a	63.3a	69.6a	58.3a	60.6a
FeHI (%)	23	21b	23b	27a	22b	23a	22a	24a	14b	19b	21ab	25a	25a
MnRIE (g)	18.1	17.8a	18.3a	17.5a	19.0a	17.8b	16.5b	17.3b	25.9a	16.8a	18.3a	18.0a	18.7a
GMnC (mg kg^−1^)	3.2	2.9b	3.5a	3.4a	3.0b	2.7c	3.7a	3.1b	3.3b	2.95c	3.03bc	3.35a	3.28ab
SMnC (mg kg^−1^)	19.0	17.3b	18.4b	19.9b	23.9a	17.4b	14.4c	19.3b	30.2a	16.8b	19.4ab	19.1ab	19.8a
MnHI (%)	16.0	15bc	17ab	18a	14c	14c	20a	16b	11d	16a	15a	17a	16a
CuRIE (g)	5.3	5.6a	5.4a	5.1a	4.9a	6.7a	5.5b	4.6c	6.0ab	4.5b	5.4a	5.4a	5.7a
GCuC (mg kg^−1^)	1.4	1.39b	1.43b	1.68a	1.26b	1.61a	1.38b	1.40ab	1.34b	1.24b	1.27b	1.57a	1.55a
SCuC (mg kg^−1^)	5.0	4.9a	5.0a	4.9a	5.5a	6.0a	4.7b	4.5b	6.4a	4.0b	5.2a	5.1a	5.4a
CuHI (%)	24	23b	23b	29a	22b	21b	22b	27a	19b	26a	21b	26a	24ab

n^a^: number of observations. Means in a row followed by different lowercase letters are significantly different at different yield ranges, cultivars and N levels (P<0.05).

**Table 3:** Correlative coefficients (r) among the measured parameters at maturity (n = 149).

	ZnRIE		FeRIE		MnRIE		CuRIE		GY
GY	−0.58***	GY	−0.20*	GY	ns	GY	−0.18*	GZnC	−0.36***
SY	ns	SY	ns	SY	0.41***	SY	0.30***	GFeC	ns
GZnC	0.54***	GFeC	ns	GMnC	ns	GCuC	0.29***	GMnC	0.22**
SZnC	0.90***	SFeC	0.89***	SMnC	0.78***	SCuC	0.76***	GCuC	ns
ZnHI	−0.85***	FeHI	−0.77***	MnHI	−0.79***	CuHI	−0.58***	—	—

GY: grain yield based on 15.5% of moisture (Mg ha^−1^); SY: straw yield based on dry weight (Mg ha^−1^); ZnRIE, FeRIE, MnRIE and CuRIE: reciprocal internal efficiencies (g micronutrient requirement per Mg grain yield); GZnC, GFeC, GMnC and GCuC: grain micronutrient concentrations (mg kg^−1^); SZnC, SFeC, SMnC and SCuC: straw micronutrient concentrations (mg kg^−1^); ZnHI, FeHI, MnHI and CuHI: micronutrient harvest indices (%).

*Significant at P<0.05.

**Significant at P<0.01.

***Significance at P<0.001.

ns: not significant at P<0.05.

**Figure 1:**
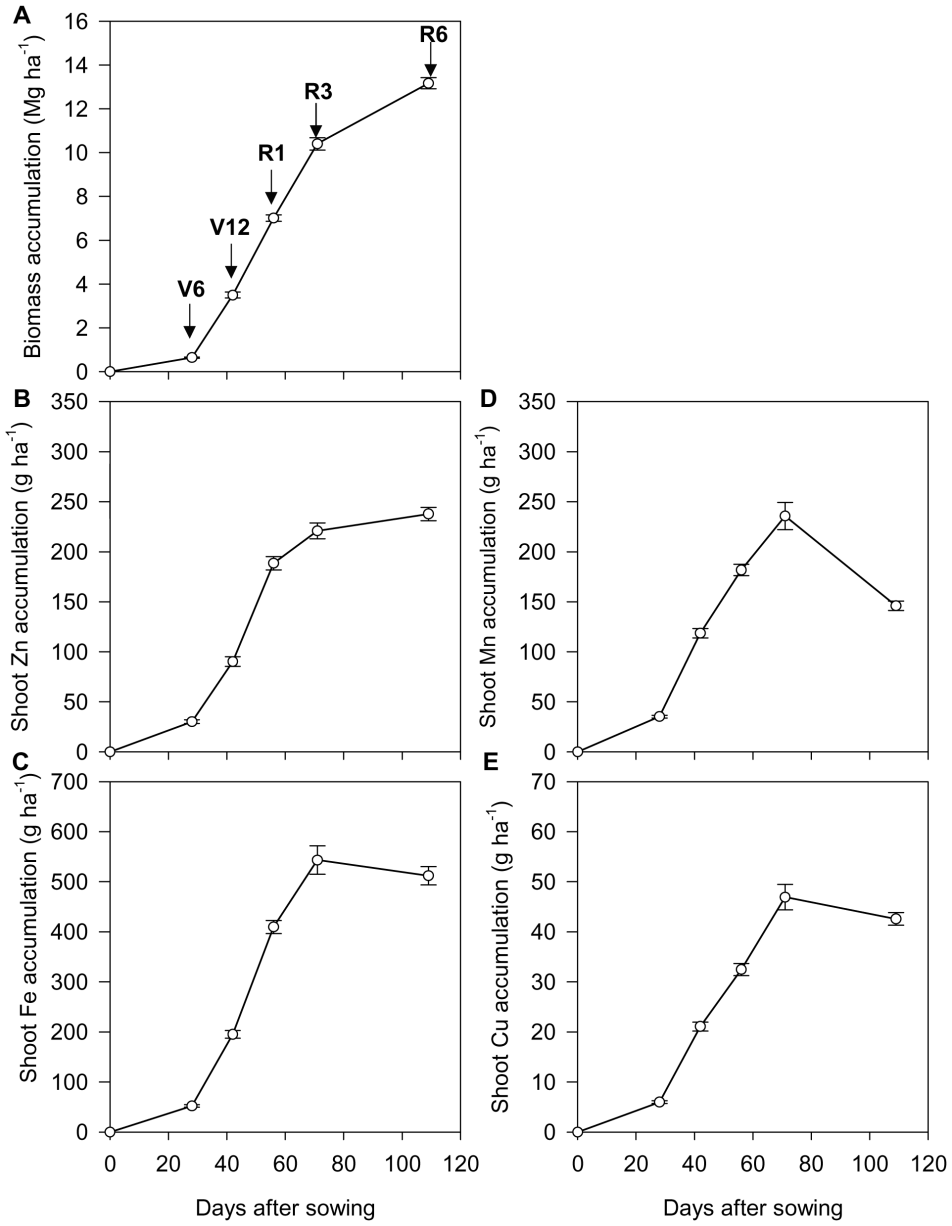
Dynamics of biomass (A), shoot Zn accumulation (B), shoot Fe accumulation (C), shoot Mn accumulation (D) and shoot Cu accumulation (E) of summer maize at V6 (six-leaf stage, n = 70), V12 (12-leaf stage, n = 54), R1 (silk emerging, n = 115), R3 (milk stage, n = 81) and R6 (physiological maturity, n = 149) stages, respectively. The bars represent the standard error of the mean.

Overall, seasonal biomass production and shoot Zn accumulation continued to increase throughout the growing season. Seasonal accumulation of shoot Fe and Cu showed a slight decrease from R3 to R6 while shoot Mn accumulation showed a large decrease from R3 to R6 (Figure 1 and Figure S1). At silking stage (R1), more than three quarters of shoot Zn, Fe and Cu accumulation, and more than 100% of shoot Mn accumulation, had occurred compared to only half of the biomass accumulation (Figure 1 and Figure S1). Generally, throughout the growing season, the biomass and shoot micronutrient accumulation, as a percentage of the total (at maturity) decreased in the order Mn>Cu>Fe≥Zn>biomass (Figure S1).

During the growth stages, the highest accumulation rates of biomass and shoot Fe and Zn consistently occurred between V12 and R1. During this period, biomass accumulated 3.5 Mg ha^−1^ with an average growth rate of 251 kg ha^−1^ d^−1^; shoot Zn accumulated 98 g ha^−1^ with an average accumulation rate of 7.0 g ha^−1^ d^−1^; shoot Fe accumulated 215 g ha^−1^ with an average accumulation rate of 15.3 g ha^−1^ d^−1^. In contrast, the highest accumulation rates of shoot Mn (83 g ha^−1^ in total, with an average accumulation rate of 6.0 g ha^−1^ d^−1^) and Cu (15 g ha^−1^ in total, with an average accumulation rate of 1.1 g ha^−1^ d^−1^) occurred between V6 and V12 (Figure 1).

### Relationships between yield and shoot micronutrient accumulation in response to different yield ranges

To understand the relationship between yield and shoot micronutrient accumulation in relation to grain yield, all the data in this study was grouped into 4 yield ranges as follows: <7.5 Mg ha^−1^ (the number of observations, n = 58, mean yield 5.9 Mg ha^−1^, recorded as GY1), 7.5–9.0 Mg ha^−1^ (n = 45, mean yield 8.2 Mg ha^−1^, recorded as GY2), 9.0–10.5 Mg ha^−1^ (n = 26, mean yield 9.7 Mg ha^−1^, recorded as GY3) and >10.5 Mg ha^−1^ (n = 20, mean yield 11.4 Mg ha^−1^, recorded as GY4) (Table 1). As shown in Table 2, the increase in grain yield from GY1 to GY2 was mainly attributed to thousand grain weight (an increase of 12%) and grains per ear (an increase of 11%). The increase in grain yield from GY2 to GY3 was mainly attributed to grains per ear (an increase of 14%) and HI (which increased from 50% to 53%). The further increase in grain yield from GY3 to GY4 was mainly attributed to HI (which increased from 53% to 55%).

With the increase of grain yield from GY1 to GY4, ZnRIE decreased gradually from 36.3, 32.4, 26.6 to 18.0 g. However, FeRIE (ranging from 57.4 to 68.6 g), MnRIE (ranging from 17.5 to 19.0 g) and CuRIE (ranging from 4.9 to 5.6 g) were not significantly affected by yields (Table 2). With increasing yield from GY1 to GY3, grain Zn concentration (average 16.9 mg kg^−1^) was not affected, but it was significantly decreased to 12.2 mg kg^−1^ when grain yield increased from GY3 to GY4. Similar results were found for grain Fe concentration. Straw Zn concentration showed a decreasing trend from 24.4 to 10.7 mg kg^−1^ while straw Mn concentration showed an increasing trend from 17.3 to 23.9 mg kg^−1^ with the increasing yield from GY1 to GY4. In contrast, straw Fe and Cu concentrations were less affected by yields. Grain Mn concentration was significantly higher in GY2 and GY3 than GY1 and GY4. Similarly, grain Cu concentration was the highest in GY3 than the other three yield ranges (Table 2). With an increasing grain yield from GY1 to GY4, ZnHI increased gradually from 41%, 48%, 54% to 60% while FeHI (ranging from 21% to 27%), MnHI (ranging from 14% to 18%) and CuHI (ranging from 22% to 29%) were the highest in GY3 than the other three yield ranges (Table 2).

The response of ZnRIE to increasing grain yield could be classified into two response stages. When grain yield was increased from GY1 to GY3, ZnRIE decreased from 36.3 g to 26.6 g (a decrease of 27%) because of increasing ZnHI (which increased from 41% to 54%) and significantly declining straw Zn concentration (a decrease of 30%) coupled with the relatively constant grain Zn concentration (average 16.9 mg kg^−1^) (Table 2). With the increasing yield from GY3 to GY4, ZnRIE further declined from 26.6 to 18.0 g (a decrease of 32%) mainly because of a further increasing ZnHI from 54% to 60% and the decreasing Zn concentrations in both straw (which decreased by 38%) and grain (which decreased by 26%) (Table 2). Furthermore, across all the grain yield ranges, ZnRIE was significantly positively correlated with Zn concentrations of grain (r = 0.54***) and especially straw (r = 0.90***) but negatively associated with grain yield (r = −0.58***) and especially ZnHI (r = −0.85***) (Table 3). Similarly, FeRIE, MnRIE and CuRIE were consistently positively associated with their respective straw concentrations and straw yields (for MnRIE and CuRIE) but negatively correlated with their respective harvest index and grain yield (for FeRIE and CuRIE) (Table 3).

**Figure 2:**
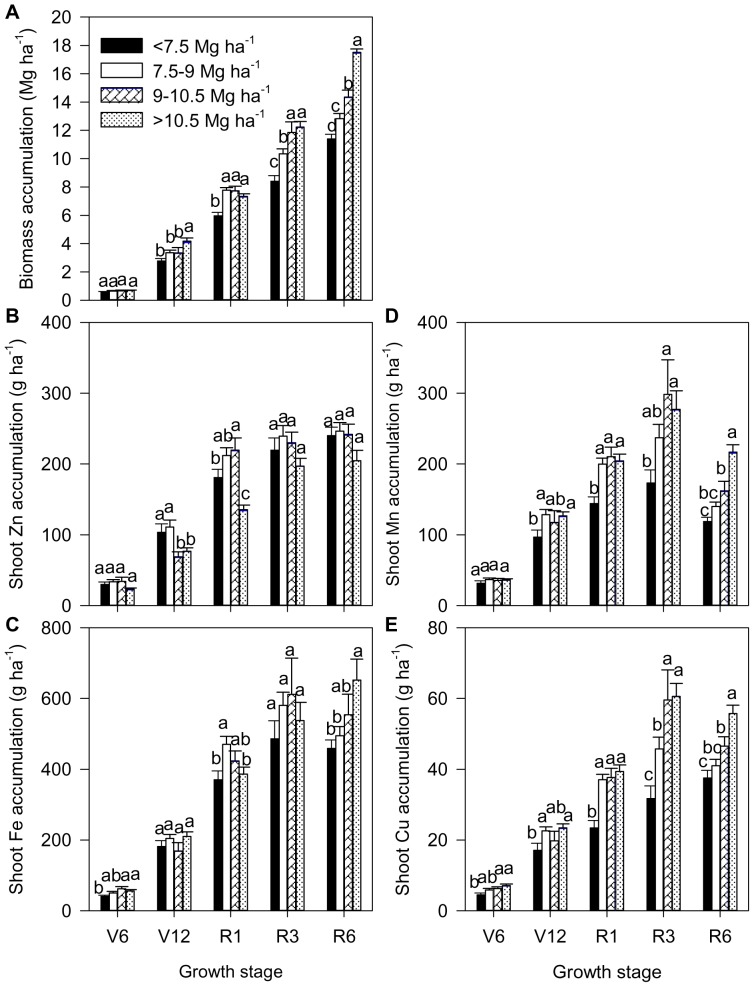
Dynamics of biomass (A), shoot Zn accumulation (B), shoot Fe accumulation (C), shoot Mn accumulation (D) and shoot Cu accumulation (E) of summer maize at V6 (six-leaf stage), V12 (12-leaf stage), R1 (silk emerging), R3 (milk stage) and R6 (physiological maturity) stages, respectively, with different yield ranges. The number of observations at each stage was shown in Table S2. The bars represent the standard error of the mean. Bars with different lowercase letters are significantly different at different yield ranges (P<0.05).

**Figure 3:**
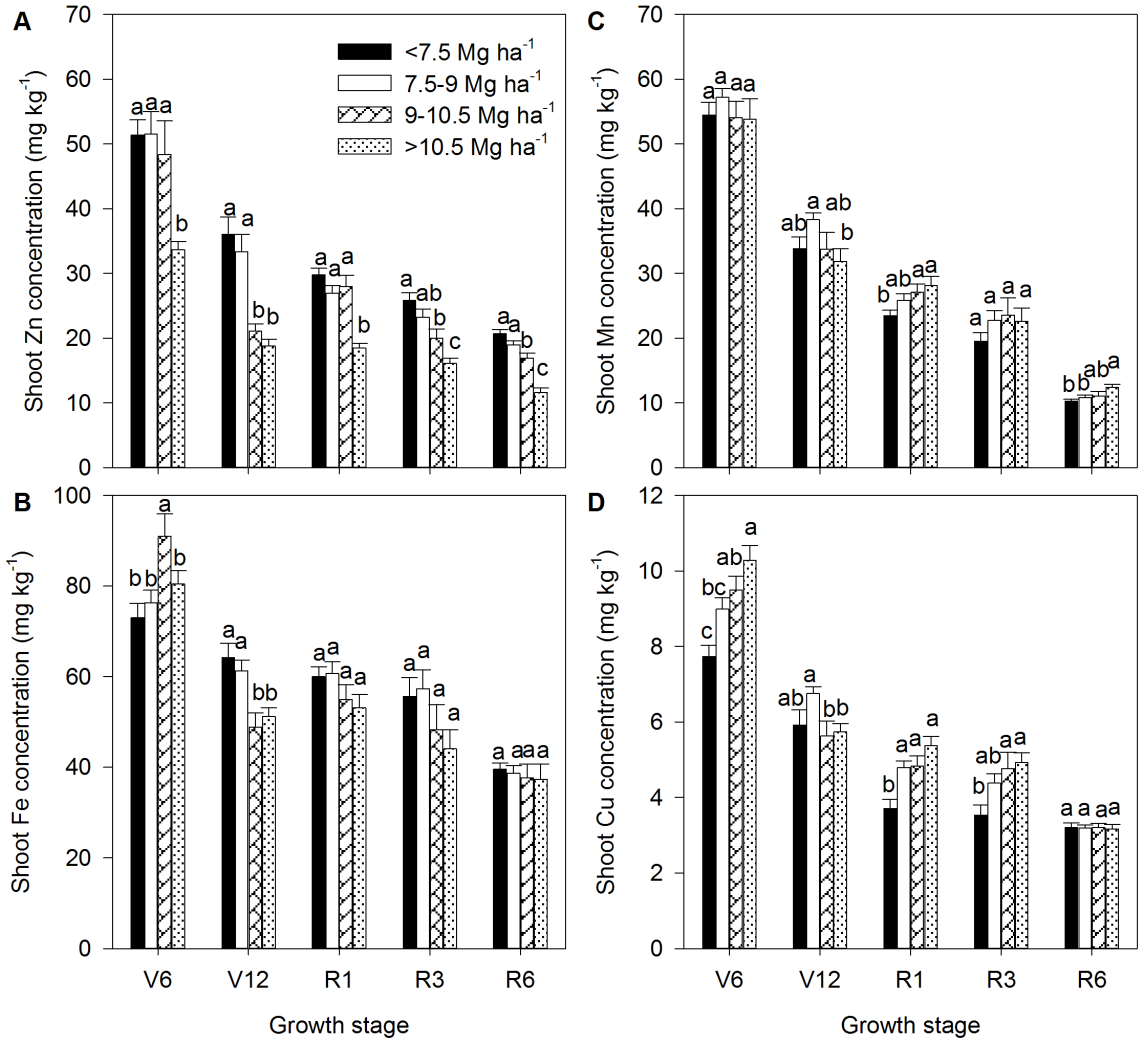
Dynamics of shoot Zn concentration (A) shoot Fe concentration (B), shoot Mn concentration (C) and shoot Cu concentration (D) of summer maize at V6 (six-leaf stage), V12 (12-leaf stage), R1 (silk emerging), R3 (milk stage) and R6 (physiological maturity) stages, respectively, with different grain yield ranges. The number of observations was shown in Table S2.The bars represent the standard error of the mean. Bars with different lowercase letters are significantly different at different yield ranges (P<0.05).

With the increase in grain yield from GY1 to GY4, the biomass production showed increasing trends, especially during the reproductive development (e.g. R3 and R6) (Figure 2A). Similar results were also found for shoot Mn and Cu accumulation due to the similar or lower shoot Mn and Cu concentrations in GY1 than the other three yield ranges, where shoot Mn and Cu concentrations were similar during the reproductive development (Figure 2D, E and Figure 3C, D). Shoot Fe accumulation among the four yield ranges were similar at V12 and R3 and tended to increase at V6 and especially at R6 with the increasing grain yield (Figure 2C). However, shoot Zn accumulation in GY4 was slightly (e.g. V6, R3 and R6 stages) or even significantly lower than the other two or three (e.g. V12 and R1 stages) yield ranges (Figure 2B).

In agreement with the overall trends shown in Figure S1, seasonal biomass production and shoot Zn accumulation continued to accumulate throughout the growing season irrespective of yield ranges (Figure 2B and Figure S2). Shoot Fe accumulation showed a decreasing trend from R3 to R6 for GY1, GY2 and GY3 but continued to accumulate throughout the growing stages for GY4 (Figure 2C and Figure S2). Shoot Mn accumulation decreased to a great extent from R3 to R6 irrespective of yields (Figure 2D and Figure S2). Similarly, shoot Cu accumulation showed a decreasing trend from R3 to R6 except GY1 (Figure 2E and Figure S2).

According to the increased grain yield from GY1 to GY4, nearly 75%, 86%, 91% and 66% of pre-silking shoot Zn accumulation, 81%, 95%, 76% and 59% of pre-silking shoot Fe accumulation, 62%, 90%, 81% and 71% of pre-silking shoot Cu accumulation and more than 100% of pre-silking shoot Mn accumulation occurred compared to 52%, 61%, 54% and 42% of pre-silking biomass accumulation (Figure 2 and Figure S2).

### RIEs in response to different cultivars

To understand micronutrient RIEs in relation to different cultivars, all data were divided into four groups: YD (the number of observations, n = 30, mean yield 5.6 Mg ha^−1^), XY (the number of observations, n = 30, mean yield 8.0 Mg ha^−1^), ZD (the number of observations, n = 75, mean yield 8.5 Mg ha^−1^) and FY (the number of observations, n = 14, mean yield 10.2 Mg ha^−1^) (Table 1). Grain yield was in the order YD<XY<ZD<FY.

The effects of cultivars on ZnRIE were similar to those of yield ranges (described above). ZnRIE decreased gradually from 41.0, 36.1, 26.6 to 22.0 g for YD, XY, ZD and FY, respectively. The response of ZnRIE to cultivars could also be classified into two response stages. ZnRIE decreased from 41.0 g for YD to 26.6 g for ZD (a decrease of 35%) because of significantly increasing ZnHI (from 37% to 55%) and declining straw Zn concentration (a decrease of 45%) coupled with the relatively constant grain Zn concentration (average 16.7 mg kg^−1^) (Table 2). ZnRIE further decreased from 26.6 g for ZD to 22.0 g for FY (a decrease of 17%) mainly because of decreasing Zn concentrations in both grain (which decreased by 17%) and straw (which decreased by 14%). FeRIE (ranging from 54.4 to 97.4 g) and MnRIE (ranging from 16.5 to 25.9 g) were in the order XY≤ZD≤YD<FY. The significantly lower FeRIE in XY than FY is due to a lower grain Fe concentration (13.9 and 16.0 mg kg^−1^ for XY and FY, respectively) and a higher FeHI (22% and 14% for XY and FY, respectively). CuRIE was in the order ZD<XY≤FY≤YD (Table 2).

### Relationship between yield and shoot micronutrient accumulation in response to different N rates

To understand the relationship between yield and shoot micronutrient accumulation in relation to N management practices, all data were divided into four groups: N-0 (the number of observations, n = 30, mean yield 6.1 Mg ha^−1^), N-low (the number of observations, n = 32, mean yield 8.3 Mg ha^−1^), N-opt (the number of observations, n = 30, mean yield 8.9 Mg ha^−1^) and N-over (the number of observations, n = 57, mean yield 8.3 Mg ha^−1^) (Table 1 and Table S1). The N-0 application rate resulted in significantly lower grain yield than the other three N application rates, while the N-opt treatment resulted in the highest grain yield. Compared to N-0 treatment, the higher grain yields with N application were mainly attributed to more biomass production and grains per ear. However, average ear numbers, thousand grain weight and HI were less affected by N rates (Table 2). Similarly, ZnRIE, FeRIE and MnRIE were also less affected by N rates. CuRIE was significantly lower in N-0 treatment compared to the other three N treatments. Grain concentrations of Zn (increased from 15.2 to 17.3 mg kg^−1^), Fe (increased from 13.4 to 17.2 mg kg^−1^), Mn (increased from 2.95 to 3.35 mg kg^−1^) and Cu (increased from 1.24 to 1.57 mg kg^−1^) showed increasing trends with the increase of N applied, although there were no significant differences between the N-opt and N-over treatments (Table 2). ZnHI and MnHI were less affected by N rates. FeHI increased from 19% to 25% with the increase of N levels from N-0 to N-opt while a further increase of N from N-opt to N-over treatments did not affect it. CuHI ranged from 21% to 26% and was inconsistently affected by N treatments (Table 2).

**Figure 4:**
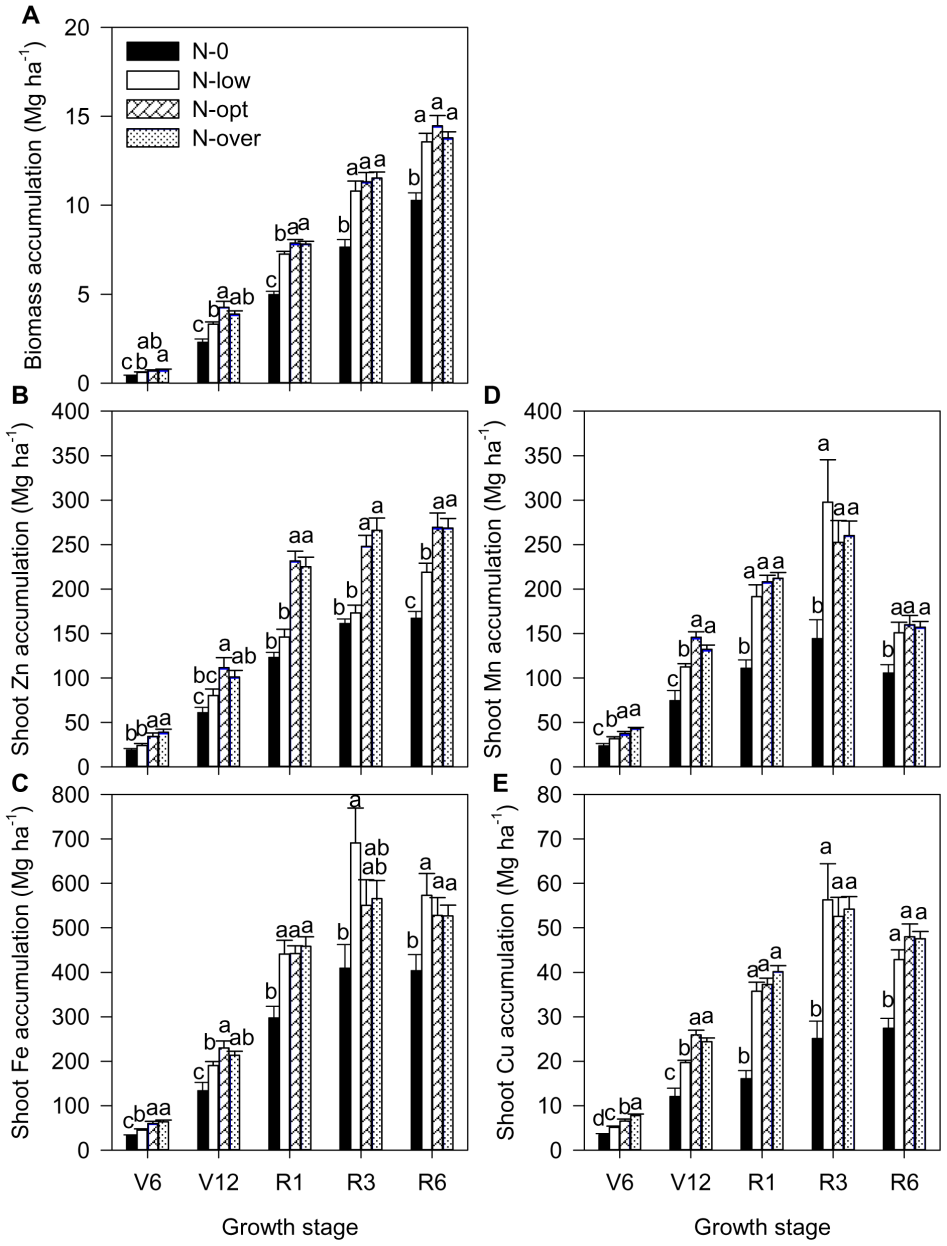
Dynamics of biomass (A), shoot Zn accumulation (B), shoot Fe accumulation (C), shoot Mn accumulation (D) and shoot Cu accumulation (E) of summer maize at V6 (six-leaf stage), V12 (12-leaf stage), R1 (silk emerging), R3 (milk stage) and R6 (physiological maturity) stages, respectively, with different N levels. The number of observations was shown in Table S2.The bars represent the standard error of the mean. Bars with different lowercase letters are significantly different at different N levels (P<0.05).

**Figure 5:**
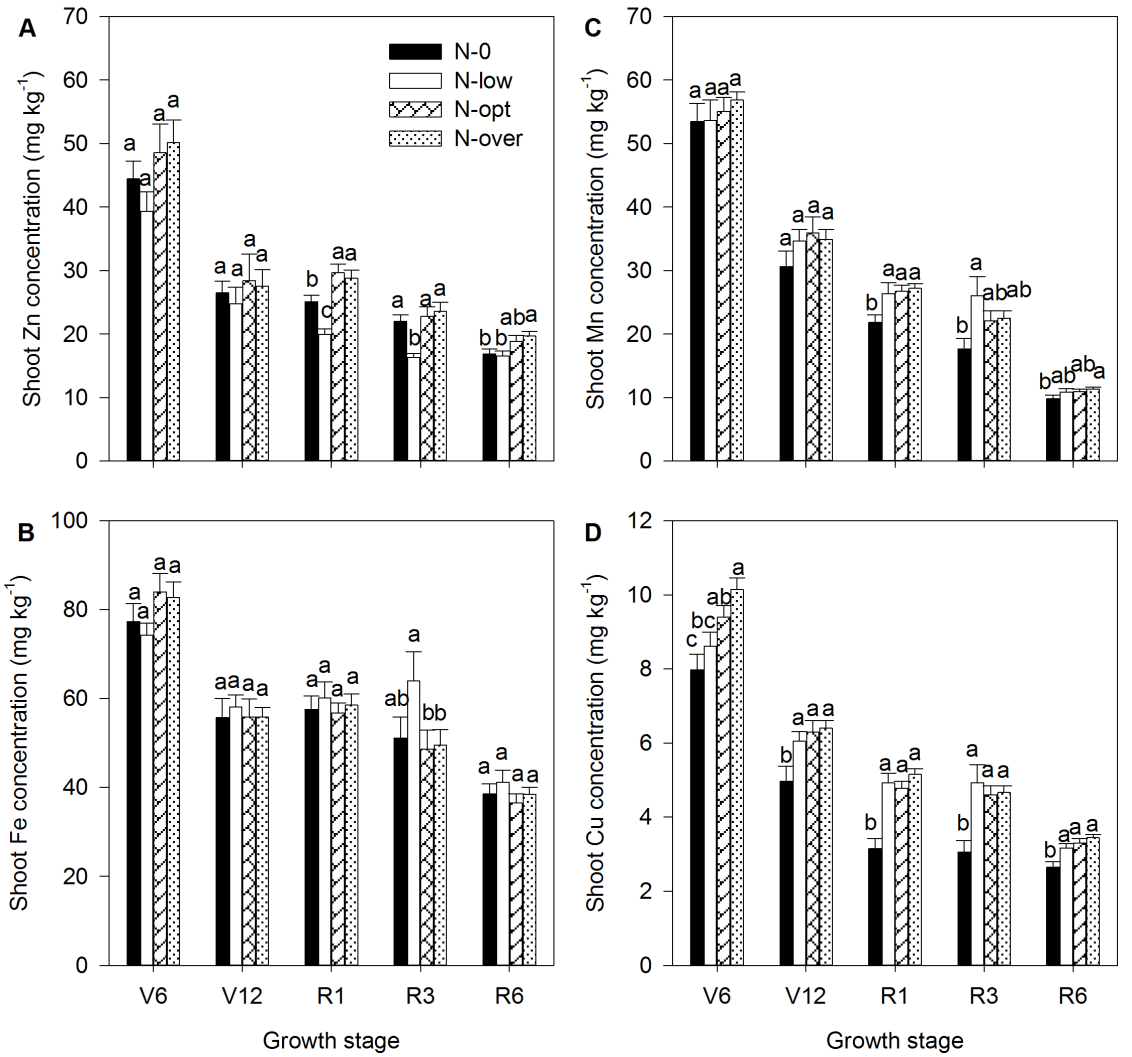
Dynamics of shoot Zn concentration (A) shoot Fe concentration (B), shoot Mn concentration (C) and shoot Cu concentration (D) of summer maize at V6 (six-leaf stage), V12 (12-leaf stage), R1 (silk emerging), R3 (milk stage) and R6 (physiological maturity) stages, respectively, with different N levels. The number of observations was shown in Table S2.The bars represent the standard error of the mean. Bars with different lowercase letters are significantly different at different N levels (P<0.05).

Before silking (R1) stage, with the increase of N levels from N-0 to N-opt treatment, the biomass production showed progressive enhancements. However, a further increasing N from N-opt to N-over treatment did not make an extra contribution to growth (Figure 4A). During the reproductive growth stages (e.g. R3 and R6), biomass production in the N-0 treatment was significantly lower than the other three N rates where there were no significant differences in biomass production (Figure 4A). Similar results were found in shoot Fe accumulation, as there were similar shoot Fe concentrations for each N rate at most of the growth stages (Figure 4C and Figure 5B). Similar results were also found for shoot Mn and Cu accumulation (Figure 4D, E). Shoot Mn and Cu concentrations were significantly lower in the N-0 treatment than the other three N treatments where shoot Mn and Cu concentrations were similar during the reproductive growth stages (Figure 5C, D). Shoot Zn accumulation tended to increase gradually with the increase of N from N-0 to N-opt throughout the growth stages, but there was a lower shoot Zn concentration in the N-low treatment compared to N-0 and N-opt treatments (Figure 4B and Figure 5A). A further increase in N from the N-opt to the N-over treatment did not affect shoot Zn accumulation as there was a similar biomass and shoot Zn concentration in both treatments (Figure 4B and Figure 5A).

With the increase of N rates from the N-0 to the N-over treatment, nearly 74%, 67%, 86% and 84% of pre-silking shoot Zn accumulation, 74%, 77%, 84% and 87% of pre-silking shoot Fe accumulation, 59%, 83%, 78% and 84% of pre-silking shoot Cu accumulation and more than 100% of pre-silking shoot Mn accumulation occurred compared to 48%, 54%, 54% and 57% of pre-silking biomass accumulation (Figure 4).

## Discussion

Overall, maize grain yield (n = 149) averaged 8.0 Mg ha^−1^ with a range from 3.9 to 12.8 ha^−1^ (Table 1), which was 49% higher than the average yields in China (5.4 Mg ha^−1^) and 61% higher than the world (5.0 Mg ha^−1^) during 2006 to 2009 [4], [32].

The relationships between grain yield and whole-plant micronutrient accumulation can be explained by studying the RIEs [19]. In this study, the average Zn, Fe, Mn and Cu RIEs were lower than corresponding values [17], [19], but ZnRIE was comparable to that from Potarzycki [33] and Bender et al. [18], indicating that RIEs varied among different environments, cultivars, management regimes (such as N management) and yields [20], [22], [23]. With the increased N rate from N-0 to N-over treatments, there was 15% (non significant) increase in ZnRIE (Table 2). Potarzycki [33] also found a slight increase in ZnRIE with an increased supply of N from 115 to 175 kg N ha^−1^. However, there was a significant 50% decrease in ZnRIE with the increase of grain yield from GY1 to GY4, although the data for GY4 excluded N-0 treatment (Tables 1 and 2). Similarly, with an increasing grain yield in the order YD<XY<ZD<FY, there was a significant 46% decrease in ZnRIE (Table 2). Furthermore, a significant negative relationship (r = −0.58***) between grain yield and ZnRIE was found (Table 3). Similarly, N reciprocal internal efficiency has also been found to decrease with increasing grain yields in spring maize and in winter wheat [22], [29]. These results indicate yields and cultivars, indeed, exert a greater influence on ZnRIE than N management practices. The substantial decrease in the ZnRIE with the increase in grain yield was predominantly associated with an increase in ZnHI and a decrease of Zn concentrations in grain and especially in straw. Cultivars showed a similar effect on ZnRIE (Table 2). An increased ZnHI with increased grain yield was also reported [19]. Behera et al. [34] reported that ZnHI was only around 30% with 6.5 Mg ha^−1^ grain yield. These results suggest grains provide a large sink for Zn translocation.

Increased biomass production may be a driving force for the uptake and assimilation of mineral nutrients such as N, P and K [15]. Similarly, our results showed, with the increases of yield and biomass production, there was a concomitant increase in the accumulation of shoot Fe, Mn and Cu at physiological maturity (Figure 2C, D, E). Furthermore, with the increase in yield from GY2 to GY4, the declining proportions of pre-silking Fe (which decreased from 95% to 59%), Cu (which decreased from 90% to 71%) and biomass production (which decreased from 61% to 42%) were consistently found (Figure 2A, C, E and Figure S2). These results indicate the significance of increasing proportions of post-silking shoot Fe and Cu accumulation, possibly because of the significantly enhanced shoot biomass production by photosynthesis during reproductive development (which increased from 39% to 58%) with increasing yields. Due to the stable or decreasing shoot Fe and Cu accumulation between R3 and R6 (Figure 2C, E and Figure S2), more supplies of Fe and Cu during the vegetative stages (V12 to R1) and reproductive stages (R1 to R3) are necessary to maximize yields. Although shoot Mn accumulation at physiological maturity increased with the increase of yield, at least 94% of pre-silking shoot Mn accumulation (Figure 2D and Figure S2) indicates that more Mn should be applied before silking, especially during V6 to V12 when the highest accumulation rate of shoot Mn generally occurred. Similarly, it has been reported that maximum Mn and Cu accumulation was achieved at stage R3, possibly due to losses during leaf senescence, leaf leaching and the low uptake rates resulting from root senescence during late-reproductive stages [19].

The similar or lower shoot Zn accumulation in GY4 compared to the other three yield ranges during all growth stages was at least in part due to the significantly lower shoot Zn concentration in GY4 compared to the other three yield ranges at each growth stage (Figure 2B and Figure 3A), probably due to a dilution effect as a result of the increased biomass production. Additionally, the shoot Zn concentration in GY4 was below the critical Zn-deficient range of 15–20 mg kg^−1^ [35], at almost all of the growing stages (Figure 3A), which indicates a potential Zn deficiency. Therefore, enhanced grain yield and biomass production may be involved in increasing Zn internal utilization efficiency (expressed by decreased ZnRIE) and Zn remobilization efficiency (expressed by increased ZnHI) at the cost of decreased Zn concentrations in straw and grain under potentially Zn deficient conditions. The higher plant Zn internal utilization efficiency in maize compared to legumes and rice was previously reported [36]. The higher Zn internal efficiency of maize with a greater yield and biomass production may be related to higher activity of carbonic anhydrase (CA) and Cu/Zn superoxide dismutase. Rengel [37] suggested that Zn-efficient wheat genotypes had an ability to maintain greater CA activity under Zn deficiency that could help maintain higher photosynthesis rates and dry matter production.

Although shoot Zn accumulation at physiological maturity was not significantly affected by the increased yield, the decreasing proportions of pre-silking shoot Zn accumulation (which decreased from 91% to 66%) together with the continued accumulation of shoot Zn throughout the growth stages with the increase of grain yield (Figure 2B and Figure S2) indicate the high reproductive-stage Zn demand. Further decreasing pre-silking accumulation of shoot Zn (48%) and Cu (45%) was found with 14.2 Mg ha^−1^ (15.5 of moisture) grain yield [18]. Grzebisz [38] also reported that vegetative tissues were only minor Zn sources while post-silking shoot Zn uptake from the soil during grain filing was the major source for growing kernels. Therefore, in order to gain higher grain yield and avoid a potential Zn deficiency, more Zn and season-long supply of Zn is necessary for maximum yields, especially during the V12 to R1 growth stages when the highest Zn accumulation rate occurs. The higher Zn and Fe accumulation rates during V12 to R1 may be because ear size and number of ovules are being established while the stalk and leaves are accumulating more photosynthates [15].

Excessive N fertilization in intensive agricultural areas of China has resulted in serious environmental problems. Currently, improving N management and developing related policies are major issues in crop production and environmental protection in China. In this study, increasing N supply from the N-0 to N-opt treatment tended to increase not only grain Zn, Fe, Mn and Cu concentrations, but also grain yields while further increased N from N-opt to N-over treatment did not result in an increase in grain concentrations but resulted in a decrease in yield (Tables 1 and 2). Similarly, there were also not any differences between N-opt to N-over treatments in shoot concentrations and the accumulation of micronutrients during any of the growth stages (Figures 4 and 5). These results indicate that optimized N management by reasonably decreasing N input was able to maintain plant nutrition of micronutrients for maximum yields as well as grain concentrations. In agreement with our results, Oktem et al. [39] previously reported the positive effects of N on Zn, Fe and Cu, but not Mn, concentrations in maize grain. Very recently, the positive effects of N on grain micronutrient concentrations (less pronounced for Zn) were also found [19]. It has been reported that application of Zn-enriched urea results in higher productivity and grain Zn concentrations in a rice–wheat cropping system than the same rate of ZnSO_4_ and urea applied separately [40], [41]. It is also convenient to apply a split application of Zn later to maintain season-long Zn supply for maize by overcoming the rapid fix on a calcareous soil because urea is often split applied. Therefore, an optimized Zn-enriched urea management strategy may be an economical method of improving Zn concentration in maize and maintaining sufficient Zn nutrition for higher grain yield.

## Conclusions and Remarks

With increases in yield and biomass production, maize shoots contained more Fe, Mn and Cu at maturity but the pre-silking proportions of shoot Fe and Cu decreased. These results indicate that with increasing yield, more Fe and Cu would be needed, not only during the vegetative stages, but also reproductive stages (e.g. R1 to R3) for maximum yields. In contrast, more Mn should be applied before silking, especially during V6 to V12 when the highest accumulation rate of shoot Mn generally occurred. Shoot Zn accumulation was non-significant among the yield ranges possibly due to a dilution effect or a potential Zn deficiency, or because of the improvements in both Zn utilization efficiency (e.g. a decrease in ZnRIE) and Zn remobilization efficiency (e.g. an increase in ZnHI). Furthermore, the substantial decrease in ZnRIE with the increase of yield was largely associated with an increase in ZnHI, at the cost of the decreased Zn concentrations in grain and especially in straw. Cultivars had the similar effects on ZnRIE. Increasing N supply generally increased micronutrient concentrations of maize grain [19], [39]. However, optimizing N management by reasonably decreasing N input did not result in any negative effects on plant and grain nutrition of micronutrients, while achieving the highest yield. These results showed that optimized N management is an applicable strategy to improve micronutrient nutrition for maximum yield.

## Supporting Information

Figure S1 Changes in biomass and micronutrient (Zn, Fe, Mn and Cu) accumulation expressed as biomass and micronutrient accumulation at each stage divided by their corresponding values at maturity. V6: six-leaf stage; V12: 12-leaf stage; R1: silk emerging; R3: milk stage; R6: physiological; the number of observations was 70, 54, 115, 81 and 149 at V6, V12, R1, R3 and R6, respectively, as shown in Table S2.
(TIF)

Figure S2 Changes in biomass and micronutrient (Zn, Fe, Mn and Cu) accumulation expressed as biomass and micronutrient accumulation at each stage divided by their corresponding values at maturity for (A) yield <7.5 Mg ha^−1^, (B) yield between 7.5 to 9 Mg ha^−1^, (C) yield between 9 to 10.5 Mg ha^−1^, (D) yield >10.5 Mg ha^−1^. V6: six-leaf stage; V12: 12-leaf stage; R1: silk emerging; R3: milk stage; R6: physiological; the number of observations was 70, 54, 115, 81 and 149 at V6, V12, R1, R3 and R6, respectively, as shown in Table S2.
(TIF)

Table S1 N split supply as urea during the vegetative period of summer maize from 2008 to 2011based on determination of soil mineral N (N_min_) of 0–90 cm at sowing, V5, V6, V10 and V12 stages in the field.
(DOCX)

Table S2 Number of observations at different growth stages with different yield ranges and N levels.
(DOCX)
