# Correction: Zinc, Iron, Manganese and Copper Uptake Requirement in Response to Nitrogen Supply and the Increased Grain Yield of Summer Maize

**DOI:** 10.1371/journal.pone.0106593

**Published:** 2014-08-25

**Authors:** The PLOS ONE Staff

## Notice of Republication

This article was republished on August 1, 2014, due to the errors in Figure 5 and Table 2. The publisher apologizes for these errors. Please download the PDF again to view the corrected article. The originally published, uncorrected article and the republished, corrected article are provided here for reference.

## Supporting Information

File S1 Originally published, uncorrected article
(PDF)

File S2 Republished, corrected article
(PDF)
