# Supplementary figures and images for: Zinc, Iron, Manganese and Copper Uptake Requirement in Response to Nitrogen Supply and the Increased Grain Yield of Summer Maize
Source: PLoS One. 2014 Apr 4;9(4):e93895. doi: 10.1371/journal.pone.0093895 (PMC3976344; doi:10.1371/journal.pone.0093895)

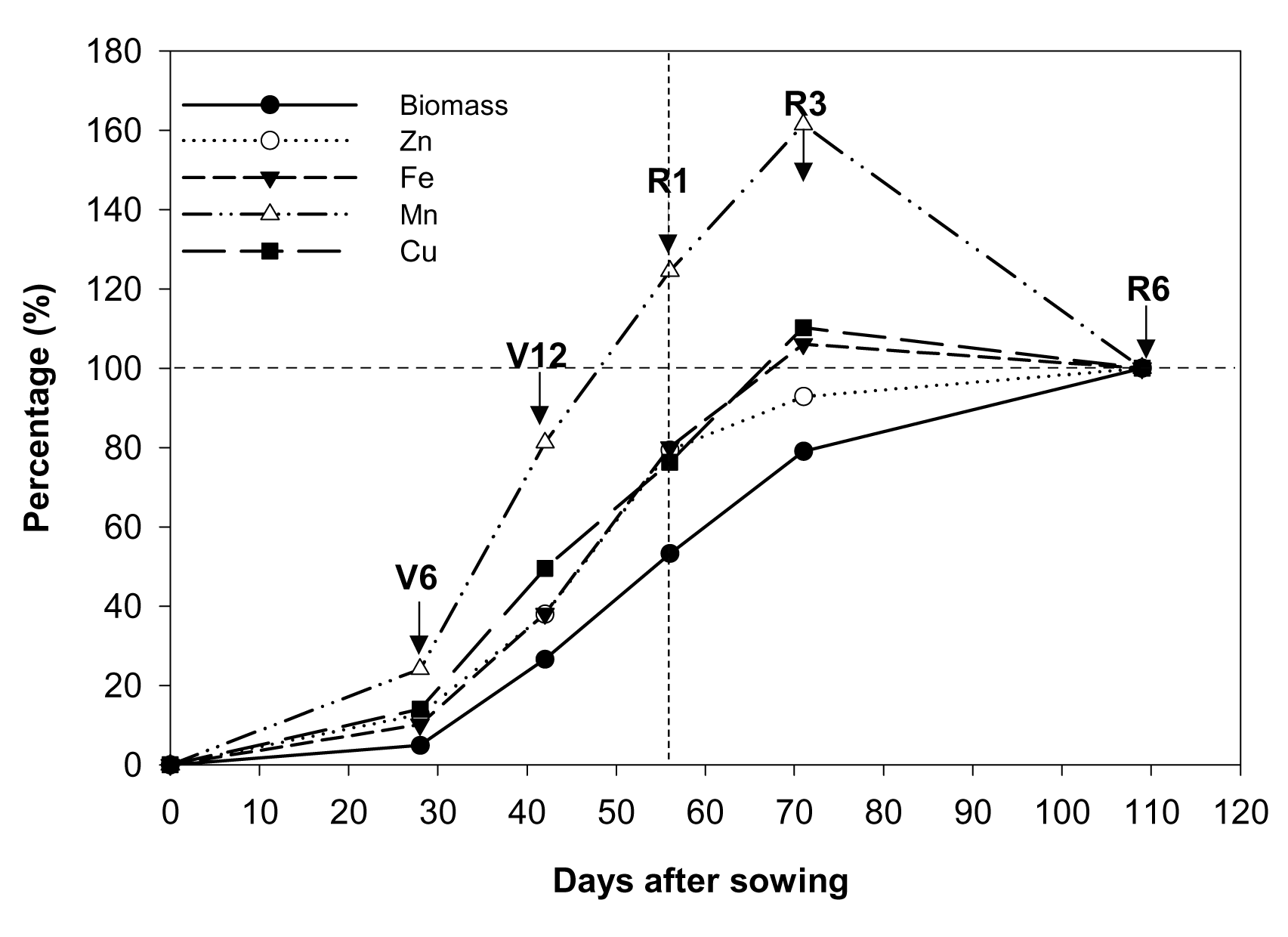

Supplement: Figure S1 — Changes in biomass and micronutrient (Zn, Fe, Mn and Cu) accumulation expressed as biomass and micronutrient accumulation at each stage divided by their corresponding values at maturity. V6: six-leaf stage; V12: 12-leaf stage; R1: silk emerging; R3: milk stage; R6: physiological; the number of observations was 70, 54, 115, 81 and 149 at V6, V12, R1, R3 and R6, respectively, as shown in Table S2. (TIF) [file pone.0093895.s001.tif]

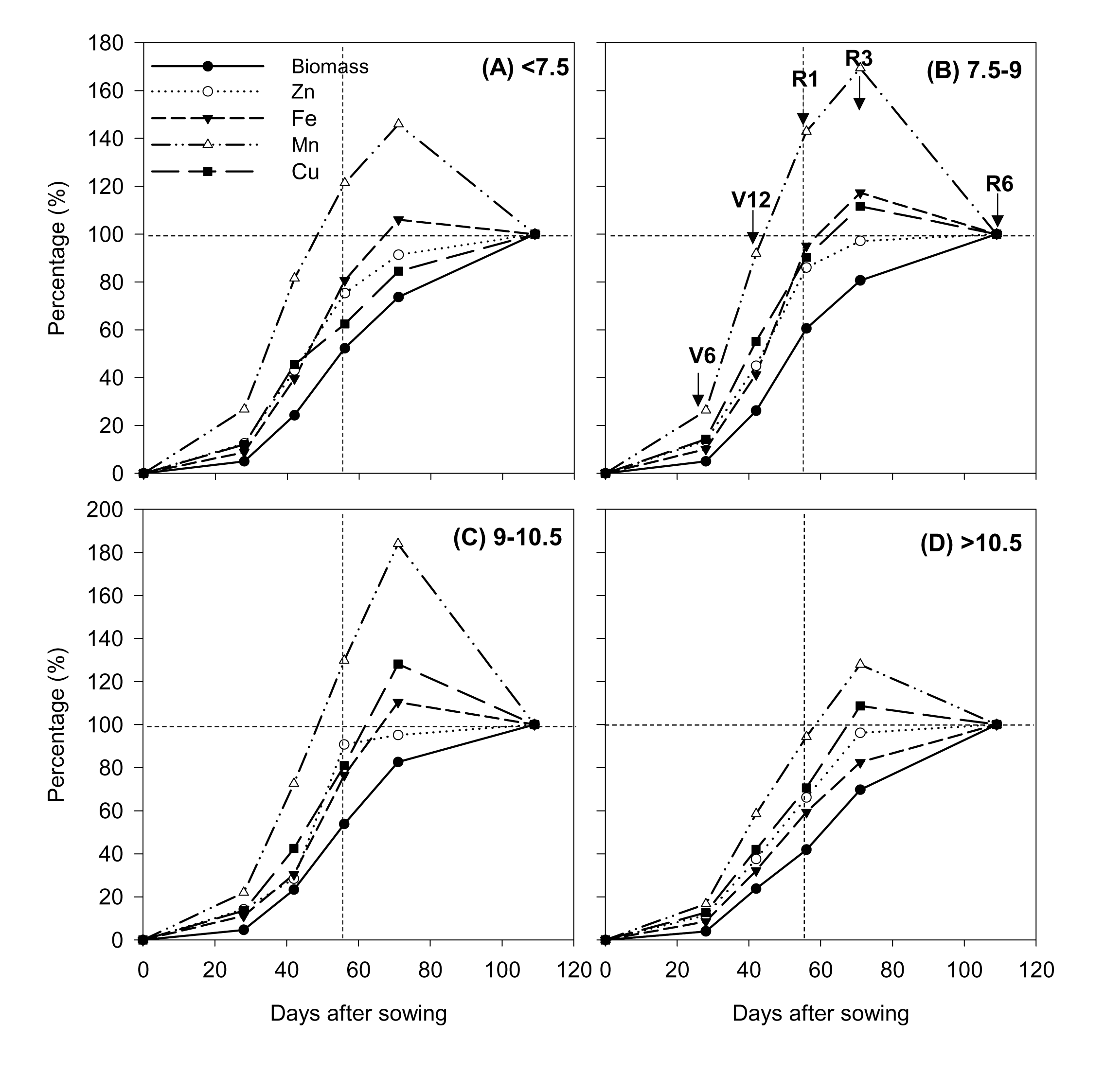

Supplement: Figure S2 — Changes in biomass and micronutrient (Zn, Fe, Mn and Cu) accumulation expressed as biomass and micronutrient accumulation at each stage divided by their corresponding values at maturity for (A) yield <7.5 Mg ha−1, (B) yield between 7.5 to 9 Mg ha−1, (C) yield between 9 to 10.5 Mg ha−1, (D) yield >10.5 Mg ha−1. V6: six-leaf stage; V12: 12-leaf stage; R1: silk emerging; R3: milk stage; R6: physiological; the number of observations was 70, 54, 115, 81 and 149 at V6, V12, R1, R3 and R6, respectively, as shown in Table S2. (TIF) [file pone.0093895.s002.tif]
