# Supplementary material for: Zinc, Iron, Manganese and Copper Uptake Requirement in Response to Nitrogen Supply and the Increased Grain Yield of Summer Maize
Source: PLoS One. 2014 Apr 4;9(4):e93895. doi: 10.1371/journal.pone.0093895 (PMC3976344; doi:10.1371/journal.pone.0093895)
Supplement: Table S1 — N split supply as urea during the vegetative period of summer maize from 2008 to 2011based on determination of soil mineral N (Nmin) of 0–90 cm at sowing, V5, V6, V10 and V12 stages in the field. (DOCX) [file pone.0093895.s003.docx]

**Table S1**

| Location | Years | Treatments | N rates |  | N amount used (kg ha^–1^) | | | | |
| --- | --- | --- | --- | --- | --- | --- | --- | --- | --- |
|  |  |  | (kg ha^–1^) |  | Base fertilizer | V5 stage | V6 stage | V10 stage | V12 stage |
| Quzhou | 2008 | N-0 | 0 |  | 0 | 0 | 0 | 0 | 0 |
|  |  | N-low | 120 |  | 22.5 | 0 | 47.5 | 50 | 0 |
|  |  | N-opt | 240 |  | 45 | 0 | 95 | 100 | 0 |
|  |  | N-over | 250 |  | 100 | 0 | 150 | 0 | 0 |
|  |  | N-over | 360 |  | 67.5 | 0 | 142.5 | 150 | 0 |
| Quzhou | 2009 | N-0 | 0 |  | 0 | 0 | 0 | 0 | 0 |
|  |  | N-low | 60 |  | 22.5 | 0 | 0 | 37.5 | 0 |
|  |  | N-opt | 150 |  | 45 | 0 | 30 | 75 | 0 |
|  |  | N-over | 225 |  | 67.5 | 0 | 45 | 112.5 | 0 |
|  |  | N-over | 250 |  | 100 | 0 | 150 | 0 | 0 |
| Quzhou | 2010 | N-0 | 0 |  | 0 | 0 | 0 | 0 | 0 |
|  |  | N-low | 74 |  | 31.5 | 0 | 21 | 21 | 0 |
|  |  | N-opt | 105 |  | 45 | 0 | 30 | 30 | 0 |
|  |  | N-over | 137 |  | 58.5 | 0 | 39 | 39 | 0 |
|  |  | N-over | 250 |  | 100 | 0 | 150 | 0 | 0 |
| Quzhou | 2011 | N-0 | 0 |  | 0 | 0 | 0 | 0 | 0 |
|  |  | N-low | 135 |  | 31.5 | 0 | 39.2 | 64.4 | 0 |
|  |  | N-opt | 193 |  | 45 | 0 | 56 | 92 | 0 |
|  |  | N-over | 250 |  | 100 | 0 | 150 | 0 | 0 |
|  |  | N-over | 251 |  | 58.5 | 0 | 72.8 | 119.6 | 0 |
| Henan | 2009 | N-0 | 0 |  | 0 | 0 | 0 | 0 | 0 |
|  |  | N-low | 120 |  | 0 | 40 | 0 | 0 | 80 |
|  |  | N-low | 180 |  | 0 | 60 | 0 | 0 | 120 |
|  |  | N-opt | 240 |  | 0 | 80 | 0 | 0 | 160 |
|  |  | N-over | 360 |  | 0 | 120 | 0 | 0 | 240 |
