# Supplementary material for: Zinc, Iron, Manganese and Copper Uptake Requirement in Response to Nitrogen Supply and the Increased Grain Yield of Summer Maize
Source: PLoS One. 2014 Apr 4;9(4):e93895. doi: 10.1371/journal.pone.0093895 (PMC3976344; doi:10.1371/journal.pone.0093895)
Supplement: Table S2 — Number of observations at different growth stages with different yield ranges and N levels. (DOCX) [file pone.0093895.s004.docx]

**Table S2**

|  | V6 | V12 | R1 | R3 | R6 |
| --- | --- | --- | --- | --- | --- |
| Total | 70 | 54 | 115 | 81 | 149 |
|  |  |  |  |  |  |
| Yield ranges (Mg ha^–1^) | | | | | |
| <7.5 | 17 | 13 | 43 | 27 | 58 |
| 7.5-9 | 19 | 13 | 31 | 21 | 45 |
| 9-10.5 | 15 | 9 | 21 | 13 | 26 |
| >10.5 | 19 | 19 | 20 | 20 | 20 |
|  |  |  |  |  |  |
| N levels |  |  |  |  |  |
| N-0 | 15 | 11 | 30 | 20 | 30 |
| N-low | 17 | 13 | 17 | 13 | 32 |
| N-opt | 15 | 11 | 30 | 20 | 30 |
| N-over | 23 | 19 | 38 | 28 | 57 |
